# p53 Protein Stability Plays a Crucial Role in NaB-Mediated Apoptosis in Colorectal Cancer Cells

**DOI:** 10.3390/cimb47080579

**Published:** 2025-07-22

**Authors:** Jeong Yeon Lee, Hyunju Kim

**Affiliations:** Department of Medicinal Biotechnology, College of Health Sciences, Dong-A University, Busan 49315, Republic of Korea

**Keywords:** colorectal cancer, sodium butyrate, p53, apoptosis, protein stability

## Abstract

Colorectal cancer (CRC) is associated with factors such as an unhealthy diet, physical inactivity, obesity, diabetes, and chronic inflammatory conditions like inflammatory bowel disease (IBD), as well as *TP53* mutations, which are observed in a broad spectrum of CRC. Additionally, alteration in the composition of the gut microbiome community and metabolism plays a significant role in the development of colorectal cancer and its therapeutic effects. It is well known that treatment with sodium butyrate (NaB), an intestinal microbial metabolite, can induce apoptosis by activating histone deacetylase (HDAC) in cancer cells. Therefore, this study examined the relationship between NaB-induced apoptosis and p53 protein level in colorectal cancer cells. Treatment with NaB triggered cell death in the HCT116 cell line. Furthermore, a notable elevation in p53 protein level was detected following treatment with a high concentration of NaB, compared to both the control group and the low concentration NaB. Furthermore, apoptotic cell death was diminished in a p53-deficient cell line (HCT 116 p53^−/−^) and p53 protein expression was more stabilized. Although p53 mRNA expression was not affected, acetylation of p53 protein was clearly observed by high concentration NaB treatment. To demonstrate the relationship between p53 acetylation and cell death, HT29 cells were treated with a high concentration of NaB. In HT29 cells with a mutation in the p53 gene, increased cell viability, overproduction p53 protein, and hyperacetylation of p53 were observed compared to the control. The results of this study suggest that p53 protein expression plays an important role in the effectiveness of therapy utilizing gut microbiota metabolites.

## 1. Introduction

According to the World Health Organization (WHO), colorectal cancer (CRC) is known to have the fourth highest incidence rate and the second highest mortality rate among cancers [1]. The onset of CRC is associated with various factors, including chronic inflammatory bowel disease (IBD) [2], smoking [3], lack of physical activity [4], consumption of processed foods [5], obesity [6], diabetes [7], and genetic factors [8]. The progression of CRC into malignant tumors is influenced by genetic alterations in both tumor suppressor genes and oncogenes. Approximately 60% of CRC patients have mutations in the tumor suppressor *TP53* gene encoding the protein p53 [9]. Either loss of function (LOF) or gain of function (GOF) of p53 plays a role in tumor development, progression, growth, and metastasis [10,11]. In addition, p53 mutations may confer resistance to systemic therapy. Thus, understanding the role of p53 is important.

Intestinal microbiotas play a crucial role in maintaining health by being involved in metabolism, endocrine function, and immune response [12]. However, dysbiosis, an imbalance in intestinal microbial communities, has been reported to disrupt intestinal metabolism and function, leading to the development of diseases [13,14,15]. Some studies have reported that certain intestinal microbiota, such as *Bacteroides fragilis* [16], *Clostridium* [17,18], and *Fusobacterium* [19,20] can induce inflammation in the gut and contribute to the development of CRC [21]. Furthermore, intestinal microbiota can impact a host’s health through fermentation of dietary components such as starches, leading to the production of various metabolites, including short-chain fatty acids (SCFA) such as butyrate, acetate, and propionate. They serve a protective role in sustaining the colon’s health. Butyrate, in particular, is reported to suppress the development and advancement of CRC [22,23]. Butyrate can bind to G-coupled protein receptor 41 (*GPR41*, also termed FFAR3), G-coupled protein receptor 43 (*GPR43*, also termed FFAR2), and G-coupled protein receptor 109A (*GPR109A*, also termed HCA2) [24]. Furthermore, butyrate is widely recognized as an inhibitor of histone deacetylase (HDACs), regulating acetylation of transcription factors and influencing gene expression and induced apoptosis in cancer cells [25].

According to recently published research, gut-derived metabolite gallic acid can switch mutant p53 to exert oncogenic effects [26]. Therefore, it is necessary to investigate the relationship between gut metabolites and p53. However, the relationship between NaB and p53 in the development and/or treatment of colon cancer has not yet been identified. So, this study was to investigate p53 protein expression and mechanisms in butyrate-induced apoptosis.

## 2. Materials and Methods

### 2.1. Cell Culture

Human colorectal cancer cell lines, HCT116 (purchased from Korea Cell Line Bank (KCLB), HCT116 p53^−/−^ (provided from Youngchun Lee, Dong-A University, Busan, Republic of Korea), and HT-29 (purchased from KCLB, Republic of Korea) were cultured in Dulbecco’s Modified Eagle’s medium (DMEM, Welgene, Gyeongsan, Republic of Korea, #LM001) supplemented with 10% heat-inactivated fetal bovine serum (FBS, Welgene, #S001) and 1% penicillin/streptomycin (P/S, Welgene, #LS202). These cells were maintained in a humidified 5% CO_2_ incubator at 37 °C.

### 2.2. MTT Assay and Cell Proliferation Assay

Cell viability was evaluated using MTT (3-(4,5-Dimethylthiazol-2-yl)-2,5-Diphenyltetrazolium bromide, Waltham, MA, USA #M6494). Cells were plated at a density of 1 × 10^4^ cells per wells in 96-well plates containing 100 µL of culture medium and treated with varying concentrations of sodium butyrate (NaB, Sigma-Aldrich, St. Louis, MO, USA, B5887): 0, 0.1, 1, 2, 5, and 10 mM. After incubating for 24 h, the medium was removed, and 100 µL of 0.3 mg/mL MTT solution was added. The cells were then incubated for 30 min at 37 °C. Following incubation, the solution was replaced with 200 µL of dimethyl sulfoxide (DMSO), and absorbance was measured at 540 nm using a Bio-Rad microplate reader.

To assess cell proliferation, cells were seeded at 1 × 10^5^ cells per well in 6-well plates and treated with NaB at concentration 0, 1, and 10 mM for time intervals of 12, 24, 36, and 48 h. At each designated time point, cells were harvested, stained with trypan blue, and quantified using a hemocytometer.

### 2.3. Colony-Forming Assay

Cells were plated at a density of 1 × 10^3^ cells/well in 6-well plates and exposed to 10 mM NaB for 24 h. After treatment, the culture medium was refreshed every 2 to 3 days. After 12 to 14 days of incubation, the medium was aspirated, and cells were gently washed once with cold Dulbecco’s phosphate-buffer saline (DPBS). The resulting colonies were fixed with chilled methanol for 15 min at room temperature (RT) and subsequently stained with 0.1% crystal violet dissolved in methanol for 3 h at RT. Following staining, the wells were washed with tap water to remove excess dye, and plates were air-dried. Colony formation was then quantified by manually counting the number of visible colonies in each well.

### 2.4. RNA Isolation, RT-PCR, and Quantitative RT-PCR (qPCR)

Cells were seeded at a density of 3 × 10^5^ cells per well in 6-well plates and treated with NaB at concentrations of 1 or 10 mM for 24 h. Total RNA was isolated using TRIzol reagnet (Takara, San Jose, CA, USA, #9109) following the manufacturer’s instructions. For each sample, 1 µg of total RNA was used to synthesize complementary DNA (cDNA) using an oligo(dT) primer (Genet bio, Daejeon, Republic of Korea, #SR-5000) and reverse transcription polymerase chain reaction (RT-PCR) was subsequently performed. Primer sequences for the genes *GPR109a*, *GPR43*, *GPR41*, and β-actin are listed in Table 1. Quantitative real-time PCR (qPCR) was carried out using SYBR Green detection chemistry (Takara, #QPK-201) on an ABI PCR system (Applied Biosystems, Waltham, MA, USA). The expression levels of target mRNA were normalized against β-actin, and relative gene expression was calculated using 2^−ΔΔ^-threshold method. Primer sequences for *TP53*, *CDKN1A*, and *ACTB* are also provided in Table 1.

### 2.5. Western Blotting

Cells were cultured in 100 mm dishes at a density of 1 × 10^6^ cells per dish and treated with NaB at 1 or 10 mM for 24 h. After treatment, cells were lysed using RIPA lysis buffer (Thermo Fisher Scientific, Waltham, MA, USA, #89900) containing a protease inhibitor cocktail (Thermo Fisher Scientific, #1862209) supplemented with protease (Thermo Fisher Scientific, #1862209) phosphatase inhibitor cocktail (Thermo Fisher Scientific, #1862495). Lysates were centrifuged at 13,000× *g* for 20 min at 4 °C, the resulting supernatants were collected. Protein concentrations were detected using a BCA assay kit (Thermo Fisher Scientific, #23225).

Equal amounts of protein are resolved by sodium dodecyl-sulfate polyacrylamide gel electrophoresis (SDS-PAGE) and transferred onto polyvinylidene fluoride (PVDF) membranes (Millipore, Burlington, MA, USA, #IPVH00010). Membranes were blocked for 1 h at RT in TBST containing 5% non-fat milk and the incubated overnight at 4 °C with the following primary antibodies: anti-FAS (Santa Cruz Biotechnology, Dallas, TX, USA, sc-715, 1:500), anti-caspase 3 (Cell Signaling, Danver, MA, USA, cs-9662, 1:200), anti-Bcl-2 (Santa Cruz Biotechnology, sc-509, 1:500), anti-Bax (Santa Cruz Biotechnology, sc-7480, 1:500), anti-γ-H2AX (Santa Cruz Biotechnology, sc-517348, 1:500), anti-p53 (Santa Cruz Biotechnology, sc-126, 1:500), anti-Acetyl p53 (Santa Cruz Biotechnology, sc-2525, 1:500), anti-Ub (Santa Cruz Biotechnology, sc-8017, 1:500), anti-GAPDH (Santa Cruz Biotechnology, sc-32233, 1:500), and anti-Actin (Santa Cruz Biotechnology, sc-1616, 1:500).

After primary antibody incubation, membranes were washed three times in TBST (10 min per wash) and then incubated with an HRP-conjugated secondary antibody for 1 h at RT. Secondary antibodies used incubated anti-mouse IgG (Cell Signaling, cs-7076, 1:3000) and anti-rabbit IgG (Cell Signaling, cs-7074, 1:3000). Detection was carried out enhance chemiluminescence (ECL) reagents (Millipore, #212185), and signals were visualized on X-ray film. Band intensities were quantified using Image J software (download at https://imagej.net/) and expressed as fold change relative to control.

### 2.6. Immune Fluorescence (IF) Staining

Cells were plated in 12-well plates at a density of 8 × 10^4^ cells per well and treated with NaB at concentration of 1 and 10 mM for 24 h. After treatment, cells were fixed with 4% paraformaldehyde (PFA) for 30 min at RT. Following fixation, cells were rinsed three times with Dulbecco’s phosphate-buffered saline (DPBS) for 5 min each and then permeabilized an blocked in solution containing 5% bovine serum albumin (BSA) and 0.1% Triton X-100. Subsequently, cells were incubated overnight at 4 °C with either γ-H2AX antibody (Santa Cruz Biotechnology, sc-517348) or p53 antibody (Santa Cruz Biotechnology, sc-126), both diluted at 1:500. After primary antibody incubation, cells were treated with fluorescence-conjugated secondary antibody (Abcam, Cambridge, UK, #ab6787) at 1:1000 dilution for 1.5 h at RT. Cells were then washed three times with DPBS and counterstained with 4′,6-diamidino-2-phenylinodole (DAPI, Thermo Fisher, Waltham, MA, USA, 1:10,000) for 10 min at RT.

Finally, cells were washed three times with DPBS and mounted with a coverslip using a fluorescence-compatible mounting medium (IBIDI GmbH, Grafefing, Germany; Carl Zeiss, Jena, Germany, #50001). Fluorescence signals were analyzed using a confocal laser scanning microscope (Carl Zeiss, #LSM700).

### 2.7. Cycloheximide (CHX) Treatment

Cells were seeded into 35 mm dishes at a density of 3 × 10^5^ cells/dish and incubated with NaB at 1 mM or 10 mM for 24 h. After NaB treatment, cycloheximide (CHX, Sigma-Aldrich, #S01810) was added to the culture medium at a final concentration of 20 µg/mL. These cells were then washed with DPBS, harvested at the indicated time points, and then subjected to Western blot using an anti-p53 antibody.

### 2.8. Annexin V-FITC Apoptosis Detection by Flow Cytometry

Cells were seeded in 6-well plates at a density of 5 × 10^5^ cells per well and treated with 10 mM NaB for 24 h. Following incubation, cells were collected and subjected to apoptosis analysis using the BD Pharmingen^TM^ FITC Annexin V Apoptosis Detection Kit I (BD Pharmingen, Franklin Lakes, NJ, USA, #556547), following the manufacturer’s instructions. In briefly, cells were washed with DPBS, stained with Annexin V conjugated to fluorescein isothiocyanate (FITC) and propidium iodide (PI) in 1× Binding buffer, and incubated at RT for 15 min. The samples were analyzed using flow cytometry on the Attune Nxt Flow cytometer (Thermo Fisher Scientific).

### 2.9. Immunoprecipitation (IP)

Cells were plated in 100 mm culture dishes at density of 1.5 × 10^6^ cells per dish and treated with 10 mM NaB for 24 h. Following treatment, cells were lysed using RIPA buffer to extract total protein. The lysates were then incubated with 1 µg of anti-Sp53 antibody for 2 h at 4°C. Subsequently, protein G plus agarose beads (Santa Cruz Biotechnology, sc-2002) were added, and the mixture was incubated overnight at 4°C to allow immunocomplex formation. After incubation, the beads were washed twice with DPBS to remove nonspecific binding. The immunoprecipitated proteins were denatured by boiling in 4 × sample buffers (SDS). The resulting samples were subjected to SDS-PAGE and analyzed by Western blot using a primary antibody against ubiquitin (Santa Cruz Biotechnology, sc-8017, 1:500).

### 2.10. Statistical Analysis

All statistical analyses were performed GraphPad Prism version 5 (Boston, MA, USA). Group comparisons were conducted using Student’s *t*-test, one-way ANOVA, or two-way ANOVA, as appropriate. Statistical significance was denoted as follows: * *p* < 0.05, ** *p* < 0.01, *** *p* < 0.001.

## 3. Results

**Figure 1:**
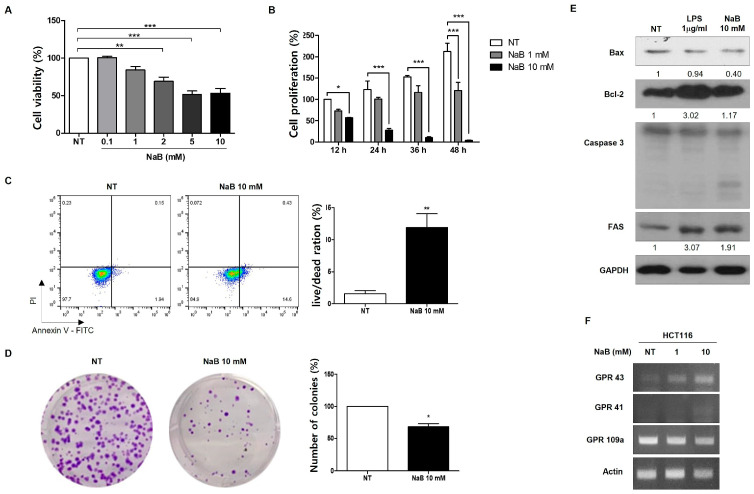
NaB induced cell death in HCT116 colorectal cancer cells. Cell death induced by NaB in a dose-dependent treatment for 24 h, as measured by MTT assay, Bar graphs are filled with darker colors depending on concentration. NT is filled with white (**A**). Cell death induced by NaB at 1- and 10-mM concentrations over a time-dependent treatment, as determined by growth curve (**B**). Apoptosis measurement after 24 h treatment with 10 mM NaB, using propidium iodide (PI) and annexin V-FITC staining, analyzed by FACs (**C**). Confirmation of colony formation after 24 h treatment with 10 mM NaB (**D**). Activation of apoptosis-related protein (Bax, Bcl-2, Caspase-3, and FAS) after treatment with 1 mg/mL LPS and 10 mM NaB for 24 h, assessed by Western blot analysis, with GAPDH as the loading control (**E**). mRNA expression of G-coupled receptors shown by RT-PCR upon NaB 1- and 10-mM treatment (**F**). * *p* < 0.05, ** *p* < 0.01, *** *p* < 0.001, calculated by *t*-test (**A**–**D**), or one-way ANOVA with Tukey test.

To investigate cell death induced by NaB treatment in colorectal cancer cells, various concentrations of NaB were added to HCT116 cells and incubated for 24 h. Cell viability was assessed using an MTT assay, which reflects the relative number of viable cells. There were no significant differences in cell viability between low-concentration NaB-treated cells and control cells. Notably, after treatment with NaB at a low concentration (1 mM), cell death was not significantly induced until 48 h. However, cell death was observed at 72 h in low-concentration NaB-treated cells (results not shown). In contrast, treatment with NaB at high concentrations (5 mM and 10 mM) induced cell death as early as 24 h (Figure 1A,B). To confirm these results, we conducted annexin-V staining and a colony-forming assay. Annexin V-FITC staining, commonly used to detect apoptotic cells, ref. [27] revealed increased death of cells treated with high concentrations of NaB compared to the control (Figure 1C). Additionally, the colony-forming assay revealed that high-concentration NaB treatment reduced the ability of cells to form colonies (Figure 1D). These findings suggest that NaB has concentration-dependent effects on HCT116 colorectal cancer cells, with high concentrations inducing cell death and low concentrations causing delayed cell death.

To investigate the mechanism of cell death, we examined expression levels of Bcl-2 family proteins and FAS to investigate various molecular mechanisms inducing cell death. Although the activities of Bcl-2 and Bax were not clearly observed with high-concentration NaB treatment, the FAS protein expression increased (Figure 1E). Additionally, it is well known that NaB affects the cellular system via G-protein-coupled receptors (GPCRs). Thus, we investigated levels of GPCRs in NaB-treated cells. *GPR43* and *GPR41* mRNA expression levels were increased in cells treated with 10 mM NaB, while *GPR109a* mRNA expression was not (Figure 1F). *GPR41* mRNA has been identified in various tissues including adipose tissue, pancreas, spleen, lymph nodes, bone marrow, and peripheral blood mononuclear cells. In contrast, *GPR43* mRNA is predominantly expressed in cells of the distal ileum, colon, and adipose tissue [28,29]. *GPR109a* expression is localized to the apical surface of colonic and intestinal epithelial cells, where it faces the lumen [30]. These expression patterns indicated that *GPR43* and *GPR109a* may play key roles in mediating the effects of high concentrations of NaB treatment in colorectal cancer cells.

**Figure 2:**
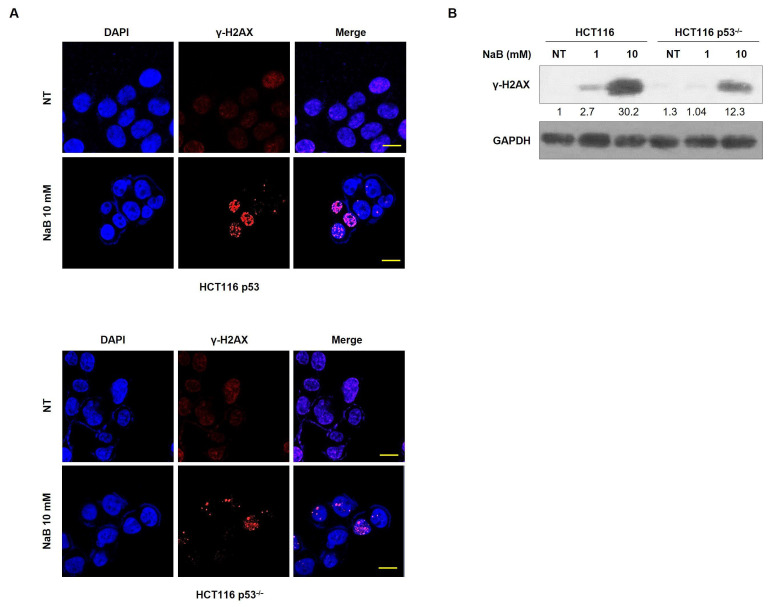
DNA damage caused by NaB treatment. Detection of DNA damage caused by NaB at 1 and 10 mM through immunohistochemistry using the γ-H2AX antibody. Scale bar = 50 μm (**A**). Activation of γ-H2AX protein expression after treatment with 10 mM NaB, determined by Western blot analysis with GAPDH as the loading control (**B**).

It is well established that severe cell injury often induces DNA double-strand breaks (DSBs), which can lead to cell death [31]. Next, we observed whether DNA damage was induced by NaB treatment. The formation of γ-H2AX foci was increased in high-concentration NaB-treated cells compared to that in low-concentration treated cells. Protein expression of γ-H2AX was also enhanced in high-concentration NaB-treated cells. However, the formation of γ-H2AX foci was not increased in HCT116 p53^−/−^ cells, in which p53 expression was deficient compared with HCT116 cells (Figure 2A,B). These results suggest that p53 expression plays an important role in DNA damage response and cell death caused by NaB treatment in colorectal cancer cells.

**Figure 3:**
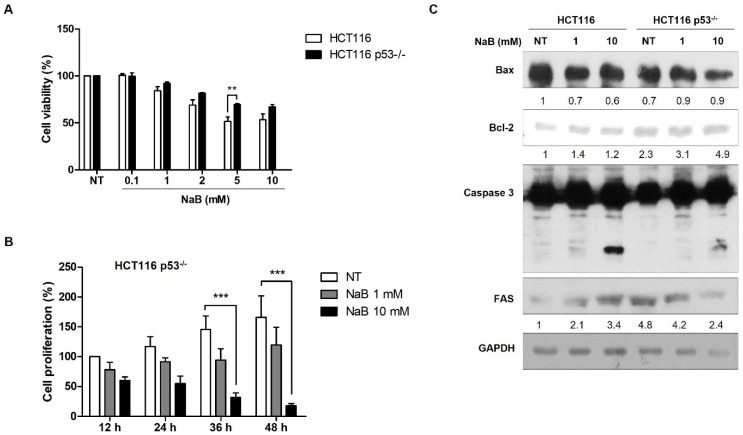
Regulation of p53 expression and cell apoptosis upon NaB treatment. Cell death induced by NaB in a dose-dependent treatment in HCT116 p53^−/−^ cells, as assessed by MTT assay (**A**). Confirmation of cell proliferation following treatment with 1 mM and 10 mM NaB in HCT116 p53^−/−^ cells, determined through a growth curve (**B**). Activation of Bax, Bcl-2, Caspase-3, and FAS protein expression in cells treated with 1 and 10 mM NaB (**C**). ** *p* < 0.001, *** *p* < 0.001, as calculated by one-way ANOVA with Turkey test.

**Figure 4:**
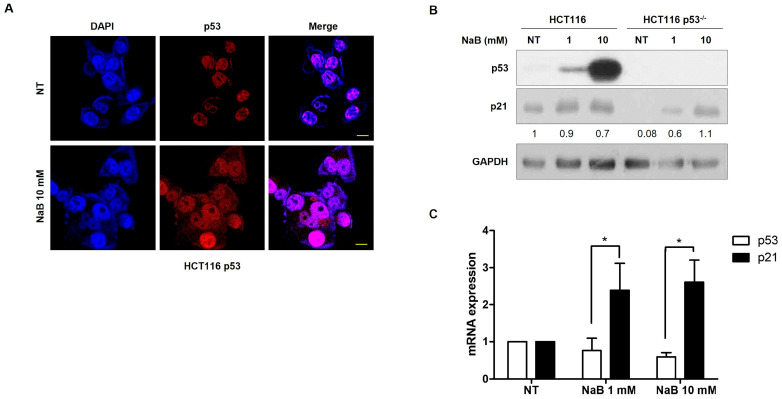
p53 protein expression by NaB treatment. Localization of p53 assessed through immunohistochemistry (IHC) using p53 antibody in HCT 116. Scale bar = 50μm (**A**). Detection of p53 protein expression by Western blot analysis in HCT116 and HCT116 p53^−/−^ cells. Scarl(**B**). An analysis of p53 and p21 mRNA expression performed through qRT-PCR in HCT116 cells (**C**). * *p* < 0.05, as calculated by one-way ANOVA with Turkey test.

The p53 tumor suppressor gene frequently exhibits mutations in many cancers. It is also known that p53 can induce apoptosis [32,33]. Therefore, we investigated the correlation between p53 expression and cell apoptosis induced by high concentrations of NaB. In HCT116 cells with the wild-type p53 protein, we observed concentration-dependent cell apoptosis. However, in HCT116 p53^−/−^ cells, which did not express p53 protein, we observed that cell apoptosis was not induced by either low or high concentration of NaB as much as HCT116 cells (Figure 3A). Furthermore, we found that apoptosis was suppressed in a time-dependent manner in HCT116 p53^−/−^ cells compared to HCT116 cells (Figure 3A,B). The induction of the FAS receptor in HCT116 p53^−/−^ cells by high concentration NaB treatment was also found to be inhibited when compared to those in HCT116 cells (Figure 3C). These results demonstrated that p53 expression is required for apoptosis by Na treatment. To assess the activity of p53 induced by NaB treatment, p53 immunostaining was performed. The immunofluorescence results showed that the presence of p53 protein is not only in cell nuclei but also in the cytoplasm in HCT116 cells. Also shown in the Western blot result, p53 protein expression was significantly increased by high concentration of NaB-treated cells. However, the p53 mRNA expression was not increased. Additionally, p21 expression was observed to be significantly increased in both HCT116 and HCT116 p53^−/−^ cells (Figure 4A,B). Although there is no effect on p53 mRNA expression from NaB treatment, the increased p53 protein suggests that it is regulated by epigenetic mechanisms such as ubiquitination, SUMOylating, or acetylation. These results explain that the increased expression of p53 protein enhanced by NaB treatment also contributes to the DNA damage response.

**Figure 5:**
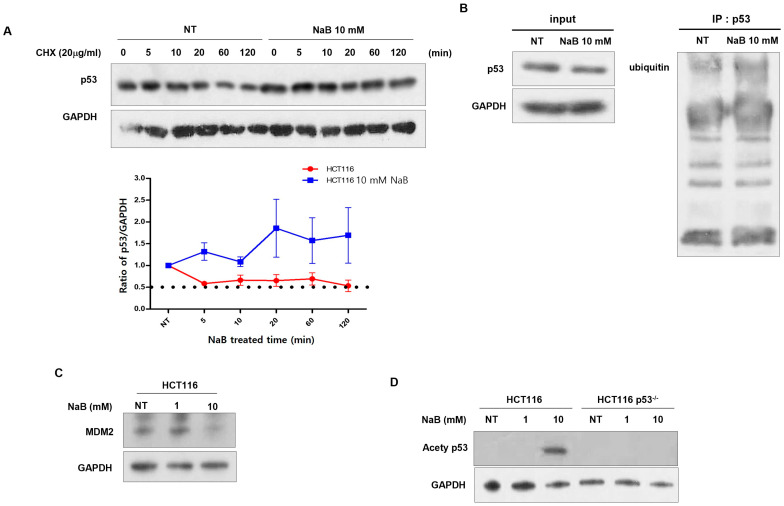
P53 protein stability via NaB treatment in HCT116 cells. Cells were treated with 20 µg/mL CHX for various time points, and the p53 expression was assessed at each time point through Western blot analysis with GAPDH as the loading control, the dot line indicates the amount of protein that is reduced by 50% (**A**). p53 ubiquitination induced by NaB treatment was measured using immunoprecipitation (IP) analysis through Western blot (**B**). Detection of mdm2 (**C**) and acetyl p53 antibody (**D**) through Western blot analysis.

**Figure 6:**
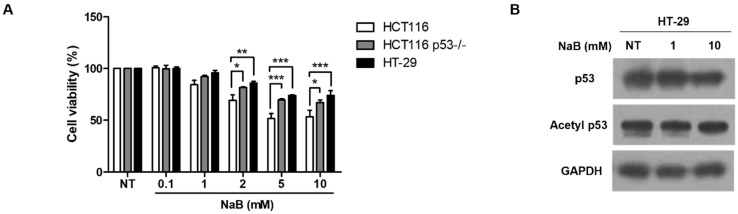
Cell death and p53 protein expression in HT-29 cells. Cell death was induced by a dose-dependent treatment of NaB for 24 h in HT-29 cells, as assessed by MTT assay (**A**). Analysis of p53 and acetyl p53 protein expression performed through Western blot (**B**). * *p* < 0.05, ** *p* < 0.01, *** *p* < 0.001, as calculated by one-way ANOVA with Turkey test.

To investigate the stabilization of p53 protein following treatment with high concentration of NaB, we measured its half-life by treating cells with cycloheximide (CHX). In HCT116 cells treated with NaB, p53 protein exhibited a half-life of approximately 5 min. In contrast, stabilization of p53 protein was observed in HCT116 p53^−/−^ cells (Figure 5A). It is known that p53 stabilization is regulated by phosphorylation, acetylation, and methylation [34,35,36]. Immunoprecipitation experiments were performed using ubiquitin (ub) antibodies. No significant differences in p53 ubiquitination were observed (Figure 5B). Also, it was observed that mdm2, an E3 ligase, was decreased after treatment with high concentration NaB in HCT116 cells (Figure 5C). However, as shown in Figure 5D, acetylation of p53 was detected following treatment with a high concentration of NaB. Thus, NaB may be involved in the acetylation of p53, thereby stabilizing the protein. Kadosh and colleagues demonstrated that the p53 mutation, R175H and R273H, occur frequently in colorectal cancer and is associated with metabolites derived from gut microbiomes [26,37]. Therefore, we treated the HT29 colon cancer cell line, which harbors a missense mutation at codon 273 in the *TP53* gene (resulting in a histidine-to-arginine substitution) and is known to overexpress p53, with NaB, and subsequently assessed cell viability. Death of HT29 cells was somewhat inhibited by high-concentration NaB compared to that of HCT116 p53^−/−^ cells (Figure 6A). We also observed increased expression of p53 protein and its hyperacetylation (Figure 6B). These findings highlight that the status of p53 protein is an important factor in apoptosis induced by gut microbial metabolites of colon cancer cells.

## 4. Discussion

Intestinal microorganisms are involved in CRC development by forming a distinct community compared to healthy microorganisms. Butyrate-producing intestinal microorganisms are prevalent across various phyla in the human colon, and sequence-based detection algorithms such as metagenomics and 16S ribosomal RNA (16S rRNA) sequencing can be employed to identify butyrate-producing microorganisms. *Faecalibacterium prausnitzii* [26,38], *Butyriivbrio crossotus* [39], and *Roseburia intestinalis* [40,41] are known as butyrate-producing species. NaB can inhibit HDACs, leading to hyperacetylation of transcription factors and the regulation of gene expression patterns in cancer. NaB also modulates cellular function through its interaction with specific cell surface receptors. As A butyrate-responsive receptor, *GPR109a* is expressed in intestinal epithelial tissues and contributes to butyrate-mediated signaling [42]. It is observed that there was no significant effect on the expression of *GPR109a* after treatment with a high concentration NaB. It has been reported that *GPR109a* expression is decreased in colon cancers and several colorectal cancer cells, regulated directly or indirectly via methylation through DNMT1. Furthermore, *GPR109a* is not associated with apoptosis induced by HDAC inhibition via butyrate [43]. Therefore, the mechanism of butyrate-induced *GPR109a* expression in colon cancer cells remains to be elucidated. It is known that NaB can induce cell death in cancer cells by regulating Wnt signaling, Bax, and p21, among others [44,45].

Butyrate is a metabolite produced by fermentation of foods rich in dietary fiber by intestinal microorganisms. Although butyrate is present at approximately 20 mM in the healthy colonic lumen, the number of butyrate-producing bacteria is significantly reduced in patients diagnosed with CRC when compared to normal individuals. This study observed the impact of butyrate concentration on cancer cells. It was noted that low concentrations of NaB had no effect on cell death, while high concentrations of NaB induced cell death. Therefore, in colon cancer treatment using gut-derived metabolites, it is necessary to increase the population of bacteria that produce butyrate or to supply a high concentration of butyrate metabolites.

Mutations in the p53 gene play a critical role in tumor development and growth. Moreover, p53 mutations are known to significantly impact a patient’s treatment response and outcome [46]. Understanding the role and mechanism of p53 in CRC has important implications for personalized treatment. Upon DNA damage, p53 serves as a cell fate determinant, controlling a range of cellular processes, including repair of damaged DNA, progression through the cell cycle, cellular differentiation, cell growth inhibition, senescence, and apoptosis [47]. In this study, we confirmed the relationship between NaB-induced p53 protein expression and cell death in colon cancer cells. It was observed that NaB increased p53 protein expression and that the absence of p53 protein inhibited the induction of cell death. Therefore, the presence or absence of p53 protein expression is a crucial factor in the treatment of cancer cells with NaB. Inducing DNA damage to inhibit the growth of cancer cells is important in cancer therapy. It is well known that p53 controls a wide range of biological activities that enable rapid cellular adaptation by coordinating various DNA damage response (DDR) mechanisms [48]. Therefore, further research on the increase in cell viability following NaB treatment in the HCT116 p53^−/−^ cell line is required in terms of DDR, DNA repair, and cell cycle. Additionally, we observed that NaB increased the stability of p53. The stability of p53 is primarily regulated by proteasome-dependent degradation through mdm2-mediated ubiquitination. When DNA is damaged, the interaction between p53 and mdm2 is disrupted, resulting in elevated p53 protein levels and transcriptional activation of p53 target genes and the induction of apoptosis [49]. Furthermore, it has been reported that acetylation of several lysine residues can enhance the transcriptional activity of p300/CBP by binding to p53 and that p300 can acetylate p53 [50]. In this study, mdm2 expression was not observed after NaB treatment. However, p53 acetylation was increased after treatment with a high concentration of NaB. These results suggest that acetylation of p53 induced by high concentrations of NaB plays a crucial role in cell death. Further research is needed to study the regulatory mechanism of p53 acetylation by NaB. Given that NaB has been reported to inhibit HDACs and the induces hyperacetylation [51], it is essential to identify the role of HDAC in the acetylation of p53. Cell death was induced by NaB in a concentration-dependent manner in p53 wild-type cells but not in the HT-29 cell line in which p53 protein was overexpressed. Hyperacetylation of p53 was observed regardless of NaB treatment. This result indicates that the effect of NaB on p53 varies depending on the cellular status. Thus, it is necessary to identify the role of p53 protein in the therapeutic effect of butyrate.

## Figures and Tables

**Table 1:** List of primers used in this study.

Gene Name	Forward	Reverse
*GPR109a*	ggacaactatgtgaggcgttgg	gggctggagaagtagtacacc
*GPR43*	gtagctaacacaagtccagtcct	ctaggtgttgctttgaagcttgt
*GPR41*	acctgctggccctggtg	ggtcaggttgagcaggagca
*TP53*	gctctgactgtaccaccatcc	ctctcggaacatctagaagcg
*CDKN1A*	tgccgaagtcagttccttgt	cattagcgcatcacagtcgc
*ACTB*	caagagatggccacggctgct	tccttctgcatcctgtcggca

## Data Availability

Human colorectal cancer cell lines, HCT116 (purchased from Korea Cell Line Bank (KCLB), HCT116 p53^−/−^ (provided from Youngchun Lee, Dong-A University, Busan, Republic of Korea), and HT-29 (purchased from KCLB, Republic of Korea). The data used to support the findings of this study are included within the article.
